# Judo exercises increase emotional expression, self-control, and psychological resilience

**DOI:** 10.3389/fpsyg.2025.1632095

**Published:** 2025-07-15

**Authors:** Mihraç Köroǧlu, Coşkun Yılmaz, Çetin Tan, Baha Engin Çelikel, Cemalettin Budak, Korhan Kavuran, Yunus Emre Susuz, Yaşar Barut, Tülay Ceylan, Fikret Soyer, Süreyya Yonca Sezer, Fatma Neşe Şahin

**Affiliations:** ^1^Faculty of Sport Sciences, Batman University, Batman, Türkiye; ^2^Kelkit Aydın Doğan Vocational School, Gümüşhane University, Gümüşhane, Türkiye; ^3^Faculty of Sport Sciences, Firat University, Elazig, Türkiye; ^4^Faculty of Sport Sciences, Erzincan Binali Yildırım University, Erzincan, Türkiye; ^5^Faculty of Sport Sciences, Bitlis Eren University, Bitlis, Türkiye; ^6^National Defense University, Ankara, Türkiye; ^7^Faculty of Health Sciences, Ondokuz Mayıs University, Samsun, Türkiye; ^8^Graduate Education Institute, Ondokuz Mayıs University, Samsun, Türkiye; ^9^Faculty of Sport Sciences, Kyrgyzstan-Türkiye Manas University, Bişkek, Kyrgyzstan; ^10^Faculty of Sport Sciences, Munzur University, Tunceli, Türkiye; ^11^Faculty of Sport Sciences, Ankara University, Ankara, Türkiye

**Keywords:** psychological resilience, sport psychology, judo, mental health, self-control, combat sports

## Abstract

**Background:**

The extant research on judo sports has principally concentrated on the physiological effects of training. Conversely, there has been limited attention paid to studies on psychological resilience, self-control, and emotional expression levels. The present study examined the effects of Judo exercises on psychological resilience, self-control, and emotional expression levels in healthy male subjects.

**Methods:**

The present study comprised 50 healthy, sedentary male subjects. The subjects were randomly divided into two groups: one group participated in judo training (JT) and the other group served as the control group (CON). The sample sizes for the JT and CON groups were both 25. The JT group underwent judo training, while the CON group maintained their usual lifestyle. The Brief Psychological Resilience Scale, the Multidimensional Brief Self-Control Scale, and the Berkeley Emotion Expression Scale were administered prior to and following the 6-week training period.

**Results:**

The study demonstrated that a 6-week judo training regimen exerted a significant effect on the psychological resilience score (e.s. = 1.047, *p* < 0.001), while no such effect was observed in the Control group (e.s. = 0.0091, *p* = 0.751). In the context of self-control levels, an effect was observed on the Initiation sub-dimension (e.s. = 1, 739, *p* < 0.001), yet no effect was found on the Inhibition dimension (e.s. = 0.052, *p* = 0.892). In the control group, a significant decrease was identified in the Initiation sub-dimension (e.s. = 0, 785, *p* = 0.001) and Inhibition sub-dimension (e.s. = 1, 861, *p* < 0.001). The findings indicate that impulse power (e.s. = 0.395) and concealment (e.s. = 0.428) exerted a negligible influence on the emotional expression sub-dimensions. Conversely, psychological resilience (e.s. = 0.886) demonstrated a substantial impact.

**Conclusions:**

The judo exercises demonstrated a favorable impact on psychological resilience, self-control, and emotional expression levels in healthy male subjects. The findings of this study may offer significant insights into the impact of judo exercises on psychological resilience, self-control, and emotion expression levels. These findings could serve as a guide for the development of future interventions and program design in the domain of sport psychology.

## Introduction

Regular physical activity has been demonstrated to have a multifaceted impact on mental and physical health, with studies showing its ability to enhance both psychological resilience and overall wellbeing (Eken et al., 2022; Kusan et al., 2025; Qiu et al., 2025; Koç et al., 2025). The evolution of judo from a martial art to an Olympic sport as a physical activity has also given rise to the need to maximize the performance of competitive athletes. In this context, Piskorska et al. (2016) emphasize that athletes' psychological and personality traits and cognitive abilities play a decisive role in performance, especially in combat sports. The unique characteristics of combat sports, including direct physical contact, pre-competition weight loss practices, the risk of pain and injury, and the constant fear of defeat, further underscore the significance of psychological and personality-based factors in determining sporting success in these disciplines (Stanković et al., 2022; Garrido-Muñoz et al., 2024). In this context, the concept of psychological resilience is of particular relevance to this sport. Psychological resilience has been defined as an important characteristic and process for athletic performance in combat sports in general (Litwic-Kaminska, 2013; Yang et al., 2019; Garrido-Muñoz et al., 2024). As a trait, it refers to a set of fixed and stable personal characteristics that enable an individual to cope with and adapt to or protect themselves from significant sources of stress or trauma (Lee et al., 2013; Garrido-Muñoz et al., 2024). The process is regarded as a dynamic response that facilitates positive adaptation to adversity (Luthar et al., 2003). In this case, the influence of personal characteristics is not fixed; rather, it varies according to the situation, the personal circumstances at that moment, and the intensity of the risk factors (Rutter, 2006). Irrespective of whether resilience is conceptualized as a process or a trait, these definitions underscore the notion that resilience signifies the capacity to confront and adeptly adapt to adversity, stress, or traumatic events, and to recuperate emotionally (Garrido-Muñoz et al., 2024).

The degree to which an individual exhibits psychological resilience is closely associated with the presence of self-control and adaptability (Rutter, 2000; Luthar et al., 2003; Epstein and Krasner, 2013; Garrido-Muñoz et al., 2024). Individuals with high resilience levels have been shown to more effectively utilize various resources to cope with challenging circumstances and maintain greater control over both their internal and external environments (Rutter, 2000; Epstein and Krasner, 2013). Psychological resilience, understood as a protective factor in the context of individual adaptive development, has been shown to minimize the physiological and psychological costs associated with coping with stress and adversity. This, in turn, ensures positive and healthy development for the individual (Epstein and Krasner, 2013). Consequently, there will be an enhancement in self-control levels (Rutter, 2000).

Self-control is generally considered to be the ability to achieve long-term, valuable goals by suppressing short-term, satisfying impulses (Hofmann et al., 2012). A growing body of research has identified that exercise programs have been shown to enhance self-control (Baumeister et al., 2007; Zou et al., 2016; Boat and Cooper, 2019). The outcomes of these programs have been demonstrated to include increased socialization, academic success, and overall quality of life. Additionally, these programs have been observed to improve mental health, promote emotional expression, and contribute to enhanced psychological wellbeing (De Ridder et al., 2012; Situ et al., 2021; Kim et al., 2022).

Another concept that contributes to psychological wellbeing is the expression of emotions (Kuzucu, 2011). Emotional expression can be defined as the process of articulating one's thoughts and feelings to another individual through various forms of communication, including speech, expressive writing, facial expressions, and gestures (Cameron and Overall, 2018; Riggio, 2017). The ability to articulate one's thoughts and emotions, which are perceived as challenges, has been demonstrated to foster the development of positive emotions and mitigate the occurrence of negative thoughts and perceived stress (Major and Gramzow, 1999; Rimé, 2007; Cameron and Overall, 2018). Concurrently, this predicament can facilitate an increased awareness of the individual's own challenges (Major and Gramzow, 1999; Kuzucu, 2011; Çarkit and Yalçin, 2018).

Studies on self-control, psychological resilience, and emotional expression in martial arts have been conducted on mixed martial arts (MMA) and Brazilian jiu-jitsu (BJJ), karate and judo players and found to have a positive effect (Domaneschi and Ricci, 2022; Potoczny et al., 2022; Invernizzi et al., 2023; de Lorenco-Lima et al., 2025). However, these studies neglected the interaction of judo training on sedentary male individuals. In this context, this study aims to make both a theoretical and methodological contribution. Due to methodological inconsistencies in the literature, the effect of judo training on self-control, psychological resilience and emotional expression levels of sedentary individuals is still unclear. The question of how self-control, psychological resilience, and emotional expression levels change in sedentary healthy male individuals in the context of judo training is sought to be answered with experimental data. The present study seeks to answer the following scientific question: Does judo training increase psychological resilience, self-control, and emotional expression in healthy sedentary men?

## Materials and methods

### Participants

The study included 50 healthy male subjects. The study was designed as a randomized, controlled experimental study. Participants were randomly assigned to two different groups: Judo group and CON group. The subjects assigned to the judo group underwent a 6-week training program focused on judo techniques, while the control group did not engage in any sport. GPower 3.1 programme was used to determine the required number of participants. The results of the power analysis sampling study showed that the study could be completed with 20 subjects in each group (effect size: 0.80; actual power: 0.89). To account for potential losses, an additional 25% was added for a total of 50 subjects, with 25 subjects allocated to each group (Control and Judo). In order to determine in which group the subjects forming the sample would be included, numbers between 1 and 50 were randomly assigned to two groups through a computerized programme (https://www.randomizer.org/). Participants with chronic or any disease were excluded from the study. Verbal and written informed consent was obtained from all participants before starting the study. The studies involving humans were approved by Approval was obtained with the decision of Munzur University, Non-Interventional Research Ethics Committee dated 27/02/2025 and numbered 2025/02. The studies were conducted in accordance with the local legislation and institutional requirements. The participants provided their written informed consent to participate in this study.

### Experimental design

**Figure 1:**
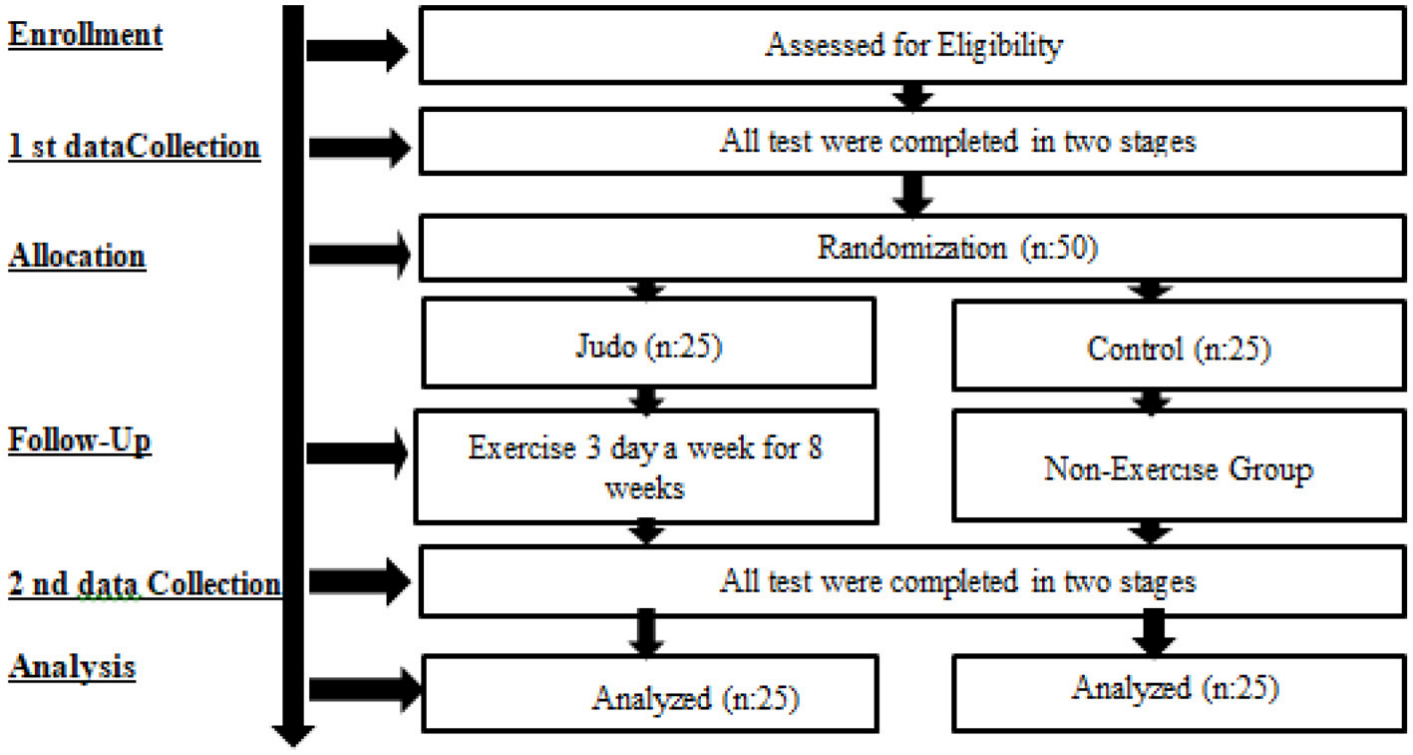
Experimental design.

Healthy male participants in the study were required to visit the laboratory environment three times. During the first visit, information about the training procedures and scales was given. Each subject received a detailed explanation of the Judo training procedure. At the next visit, which took place one week later, pre-exercise measurements were taken and values were recorded. At the end of the six-week training programme, final measurements were taken during the third and last visits (Figure 1).

### Body composition measurement

In the study, body composition was measured with Gaia 359 Plus Body-pass bioelectrical impedance analyser to indicate that the participants had similar body characteristics. The Gaia 359 Plus BodyPass was used to determine the subject's height and body weight.

The means of the participants in the study were 173.4 ± 10.54 cm, 67.04 ± 10.01 kg, 20.23 ± 1.21 years for the Judo group and 173.6 ± 9.94 cm, 71.64 ± 12.01 kg, 20.52 ± 1.47 years for the Control group (Table 1).

**Table 1:** Descriptives.

**Group**	**Height**		**Weight**		**Age**	
	**X**	**S.D**.	**X**	**S.D**.	**X**	**S.D**.
Judo	173.40	10.54	67.04	10.01	20.23	1.21
Control	173.60	9.94	71.64	12.01	20.52	1.47

### Brief psychological resilience scale

The Brief Psychological Resilience Scale (BSRS), developed by Smith et al. (2008) and adapted to Turkish culture by Dogan (2015), is a 5-point Likert-type scale consisting of six items and one sub-dimension developed to measure the psychological resilience levels of individuals. There are (2, 4, 6) reverse items in the scale. After the reverse coded items are corrected, the highest score that can be obtained from the scale can be 30 and the lowest score can be 6. A high score on the scale indicates a high level of psychological resilience. It is a scale that is easy to use and score, practical and can be applied in a short time. Dogan (2015) determined the Cronbach alpha reliability of the scale as 0.81 and the internal consistency coefficient of the scale as 0.83. As a result of these findings, it was shown that the scale is a measurement tool with high validity and reliability that can be applied in Turkish culture. In our study, the Cronbach's alpha reliability coefficient of the scale was 0.794.

### The Brief Multidimensional Self-Control Scale (BMSCS)

The scale, developed by Nilsen et al. (2020) and later adapted into Turkish by Koç et al. (2023), consists of eight items and two sub-dimensions, Inhibition and Initiation. Initiation sub-dimension consists of 2, 4, 5, and 8 items, while Inhibition sub-dimension consists of 1, 3, 6, and 7 items. Items 1, 3, 5, and 7 were reverse scored to ensure consistency and comparability. The scale is a five-point Likert type. In this study, Cronbach's alpha reliability coefficient was calculated as 0.665 for the Initiation subscale and 0.567 for the Inhibition subscale.

### Berkeley emotion expressivity questionaire

Berkeley Emotion Expressivity Questionaire (BEEQ) was developed by Gross and John (1995). The scale consists of 16 items. The scale consists of 5-point Likert-type items scored from 1 to 5 and is scored from 1 (strongly disagree) to 5 (strongly agree). The points that can be obtained from the scale vary between 16 and 80. The higher the total score obtained from the scale means that the person expresses his/her emotions behaviorally to that degree. The expression of emotions sub-dimension consists of items 1, 3, 5, 6, 10, 13, and 16; the impulse strength sub-dimension consists of items 2, 4, 7, 11, 12, 14, and 15; and the concealment sub-dimension consists of items 8 and 9. The 1st, 3rd, 8th, and 9th items of the scale are reverse scored. The Turkish adaptation of the scale was conducted by Akan and Barişkin (2017). In this study, Cronbach's alpha reliability coefficient was calculated as 0.708 for the expression of emotions subscale, 0.645 for the impulse strength subscale and 0.703 for the concealment subscale.

### Weekly training program

Judo training sessions were conducted over a period of 6 weeks, with the initial 4 weeks encompassing fundamental movements and techniques. The subsequent 2 weeks were designed to familiarize participants with combined strikes and techniques (Harrington, 2002; Broussal-Derval, 2021). Training sessions were conducted thrice weekly for a duration of 90 min, on non-consecutive days. The training intensity was maintained within the moderate to high range on the basis of the perceived exertion.

Before the study, the participants were given theoretical information about Judo; in this context, the definition, history, purpose and basic principles of Judo were explained in detail. Before starting the 6-week training program; Rei Waza (greeting), Obi Waza (belt tying), Ukemi Waza (fall techniques), Shizei Waza (stance positions), Shintai Waza (walking techniques), Kumi Kata (grips), Tai Sabaki (body turns) and Happono Kuzushi (eight-way unbalancing) were included. This preliminary preparation process was planned to allow the participants to acquire basic judo skills. Each training session lasted 90 min in total, including 20 min of general warm-up, 60 min of technical training and 10 min of cooling down. Before starting the training, an additional warm-up protocol of ~10 min was applied to activate the lumbo-pelvic-hip complex muscles of the athletes. This protocol was structured based on the method developed by Crow et al. (2012) and consisted of exercises such as double-legged bridge, side-lying hip abduction, quadrupedal lower extremity lift, gluteal rest with 60° hip flexion, dirty dog (quadrupedal hip abduction), single-legged hip extension and balance ball wall squat. Subsequent to the general warm-up, fundamental movements such as Ukemi Waza (fall techniques) and forward/backward somersaults were rehearsed for a duration of 5 min at the commencement of each training session. Furthermore, at the commencement of each session, the techniques acquired in the preceding weeks were reinforced through a brief repetition process lasting ~5 min. During the initial 2 weeks of the training program, emphasis was placed on the fundamental principles and ground techniques (ne-waza) of Judo. In the third and fourth weeks, the technical training content was structured to learn advanced techniques such as Ippon Seoi Nage, O Goshi, and Kesa Gatame. In weeks five and six, the participants engaged in a review of previously learned techniques through Uchikomi (technique review). They were also introduced to Ouchi Gari, Yakusoku Geiko, Kumi Kata at 70% intensity (with controlled resistance) and Randori (competition-like practice) at low intensity (Table 2). In Yakusoku Geiko and Randori exercises, 3-min active rest and water breaks were given between sets.

**Table 2:** Six-week judo training protocol.

**Week**	**Training content**
First and second	Shizentai: normal body posture
	Jigotai: deep defensive posture
	Ushiro ukemi: falling backward
	Yoko ukemi: falling sideways, to the right and left
	Mae ukemi: falling forward
	Ayumi ashi: overtaking each other's feet while walking
	Mae mawari ukemi
	Tsugi ashi: walking with no overtaking the feet
Third and fourth	Kumi kata: grip techniques
	Tai Sabaki
	Throwing phases
	-Kuzushi: breaking equilibrium
	-Kake: throw execution
	-Tsukuri: throwing preparation, throw entrance
	-Nage: the fall of Uke
	Training the technique of Ippon Seoi Nage and O Goshi
Fifth and sixth	Reviewing the previous techniques (Uchikomi)
	Training 0 uchi Gari
	Training the technique of Kesa- gatame
	Fighting grips (Kumi Kata)
	Yakusoku Geiko
	Fight with 70% power (Randori)

### Statistical analysis

Statistical analyses were performed via SPSS (Version 21.0 for Windows, Chicago, IL, USA) software, with the statistical significance set at 0.05. The Shapiro–Wilk normality test was performed to determine the homogeneity of the sample (Table 3). The pre-test and post-test results for each group were analyzed using a paired *t*-test. The difference values of the sub-dimension parameters of the scales used in the study were obtained by subtracting the post-test and pre-test difference values. To determine the differences between groups, a one-way analysis of variance was performed using the difference values. In addition, in the comparison of paired groups, the effect size was calculated according to Cohen's d formula [(M2 – M1)/SD pooled]. Moreover, it was interpreted as follows: 0–0.19 insignificant, 0.20–0.59 small (s), 0.6–1.19 moderate (m), 1.20–1.99 large (l), and ≥2.00 very large (v.l) (Cohen, 2013).

**Table 3:** Mean, reliability, skewness, and kurtosis values of the scales.

**(n:50)**	**Sub-dimensions**	**Number of items**	**X**	**S.D**.	**α**	***p***	**Kurtosis**	**Skewness**
Brief psychological resilience scale		6	20.39	4.6	0.794	0.004	−0.053	−0.534
The brief multidimensional self-control scale (α: 0.543)	Initiation	4	14.10	2.37	0.665	<0.001	0.462	−0.213
	Inhibition	4	12.23	2.73	0.567	<0.001	0.0819	1.228
Berkeley emotion expressivity questionaire (α: 0.561)	Expression of emotions	7	21.25	2.9	0.708	<0.001	−0.227	0.220
	Impulse power	7	24.59	4.61	0.645	<0.001	−0.747	0.919
	Concealment	2	5.06	1.83	0.703	<0.001	0.732	0.919

α, Cronbach's Alpha; X, Mean; S.D., Standard deviation.

## Results

**Figure 2:**
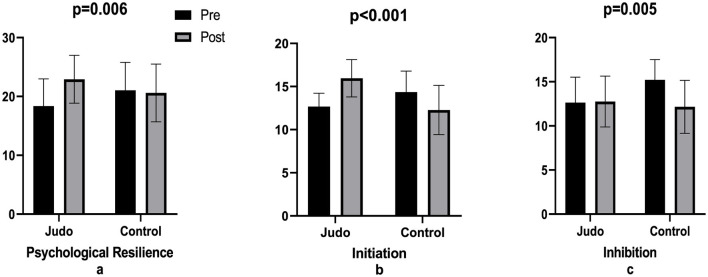
Comparison of psychological resilience and self-control levels pre- post-training. Psychological resilience **(a)**, initiation **(b)**, and inhibition **(c)**.

The study demonstrated that a 6-week judo training regimen exerted a significant effect on the psychological resilience score (e.s. = 1.047, *p* < 0.001), while no such effect was observed in the Control group (e.s. = 0.0091, *p* = 0.751) (*p* = 0.06, Table 4, Figure 2a). Judo training demonstrated a significant impact on self-control levels in the Initiation sub-dimension (e.s. = 1, 739, *p* < 0.001, Table 4, Figure 2b), while exhibiting no discernible effect in the Inhibition dimension (e.s. = 0.052, *p* = 0.892, Table 4, Figure 2c). In the control group, a significant decrease was found in the Initiation sub-dimension (e.s. = 0, 785, *p* = 0.001, Table 4, Figure 2b) and a significant increase was found in the Inhibition sub-dimension (e.s. = 1, 861, *p* < 0.001, Table 4, Figure 2c) (*p* = 0.005).

**Table 4:** Comparison of pre- and post-training group results.

	**Judo**		***d***	**Control**		***d***	***p***
	**Pre**	**Post**		**Pre**	**Post**		
P.P.	18.36 ± 4.62	22.92 ± 4.07↑	1.047^m^	21.04 ± 4.74	20.60 ± 4.92	0.091	0.006
Initiation	12.68 ± 1.55	15.96 ± 2.17↑	1.739^l^	14.36 ± 2.43	12.28 ± 2.85↓	0.785^m^	*p* < 0.001
Inhibition	12.64 ± 2.89	12.76 ± 2.88	0.052	15.20 ± 2.31	12.16 ± 3.00↓	1.861^l^	0.005
E.E.	21.44 ± 2.81	21.32 ± 2.79	0.042	21.28 ± 3.02	20.68 ± 2.78	0.207 ^m^	0.681
I.P	24.20 ± 4.92	25.80 ± 2.92	0.395^s^	26.28 ± 2.98	26.12 ± 2.42	0.059	0.159
Concealment	5.36 ± 1.75	4.68 ± 1.41	0.428 ^s^	4.96 ± 1.49	5.08 ± 1.29	0.086	0.205
E.E.T	51.00 ± 6.42	51.80 ± 3.79	0.886^m^	52.52 ± 3.15	51.88 ± 4.06	0.17	0.465

↓↑ p <0.05; P.P., psychological resilience; E.E., Emotion Expression; I.P., impulse power; E.E.T., Expression of emotions total; d., effect size; 0–0.19 insignificant, 0.20–0.59 small (s), 0.6–1.19 moderate (m), 1.20–1.99 large (l), and ≥2.00 very large (v.l).

**Figure 3:**
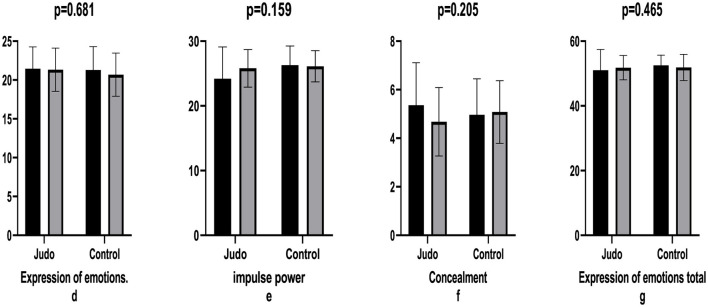
Comparison of expression of emotions levels pre- post-training. Expression of emotions **(d)**, impulse power **(e)**, concealment **(f)**, and expression of emotions total **(g)**.

The impact of judo training on various physical and psychological outcomes was examined. The findings revealed a modest effect on impulse power (e.s. = 0.395), concealment (e.s. = 0.428), and a more pronounced effect on psychological resilience (e.s. = 0.886). The expression of emotions (*p* = 681), impulse power (*p* = 159), concealment (*p* = 205), and the total expression of emotions (*p* = 465) did not demonstrate a significant difference between the groups (*p* > 0.05, Table 4, Figure 3).

## Discussion

The primary conclusions of this study indicated that a 6-week judo training regimen exhibited a substantial and robust impact on psychological resilience. Furthermore, while the study demonstrated a positive impact on the initiation sub-dimension of self-control, no significant alterations were observed in the inhibition dimension. The impact of the training variable on emotion expression levels was found to have a small effect on impulse control and concealment, and it was determined to be moderate in relation to psychological resilience. This outcome substantiated the primary hypothesis of the study, which posited that judo training enhances psychological resilience, self-control, and emotional expression levels in healthy, sedentary men. This phenomenon can be attributed to the significant role of exercise in mental health, given its positive mental and physical effects.

The findings obtained in this study reveal that judo training has positive effects on psychological resilience, self-control, and emotion expression levels of sedentary university students. A substantial body of research has been conducted on the impact of physical activity on psychological wellbeing. In a cross-sectional study conducted by Özbek and Akyüz (2022) on soccer players, it was reported that the psychological resilience levels of the participants were moderate. This finding indicates that even athletes are amenable to enhancement in terms of psychological resilience, thereby underscoring the potential of physical activity in this domain. San Román-Mata et al. (2020) posited that active engagement in health-oriented physical activities has the potential to enhance psychological resilience and emotional regulation skills, thereby reducing the prevalence of psychological distress. In a similar vein, Dunston et al. (2022) reported that individuals engaging in more intense physical activity exhibited higher levels of resilience and perseverance. Lin and Gao's (2023) study revealed a mutual interaction between physical activity and social support, indicating a collective contribution to the psychological resilience levels of young individuals. Qiu et al. (2025) also identified a substantial and favorable correlation between physical activity levels and psychological resilience in young individuals. Analogous effects were observed in the context of disparate combat sports. As reported by Yilmaz et al. (2024), a 6-week taekwondo training program led to an enhancement in self-control levels among sedentary university women. In a recent study, Jeong (2024) examined the impact of judo training on adolescents' life satisfaction and found positive results. Hami and Mohammad Hassan (2024) study indicated that a period of 8 weeks of judo training had a substantial impact on the improvement of various health and wellbeing outcomes in visually impaired veterans. These outcomes encompassed enhanced life expectancy, improved mental health, and elevated levels of competition motivation. As demonstrated by Lindell-Postigo et al. (2023), judo training has been shown to be an effective intervention for the reduction of aggressive behaviors, in addition to the enhancement of emotional intelligence and self-concept levels.

The impact of judo on individuals across diverse age groups and with unique needs is a noteworthy consideration. Yamasaki (2023) reported that judo training had positive effects on physical (e.g., gait and balance) and cognitive (memory, executive functions) functions in elderly individuals. Morales et al. (2021, 2022) have demonstrated through empirical research that adapted judo programs in children diagnosed with autism spectrum disorder (ASD) result in significant improvements across multiple domains, including motor abilities, psychosocial behaviors, social interaction, and emotional regulation. Furthermore, Križalkovičová et al. (2024) reported that judo practice can support central nervous system (CNS) maturation in young children. According to Lo et al. (2019), judo training has the potential to facilitate cognitive development in school-age children and individuals diagnosed with executive dysfunction. Furthermore, Renziehausen et al. (2022) reported that short-term adapted judo programs have the capacity to reduce stress, enhance satisfaction with the activity, and promote improvements in social relationships. Ennigkeit and Beek (2019) reported that judo increases empathy levels and reduces aggressive behaviors. Meanwhile, Sterkowicz-Przybycień et al. (2014) stated that this sport contributes to psychosocial areas such as self-discipline, serenity, effective problem solving, and socio-moral sensitivity. Biedrzycki and Laskowski (2024) underscored the notion that regular participation in judo confers a comprehensive array of benefits, encompassing both intellectual and psychosocial dimensions of wellbeing in individuals.

The rise in mental health issues (e.g., anxiety, depression, low self-esteem) among university students has been shown to have a detrimental effect on both academic performance and quality of life (Karyotaki et al., 2020; Yousif et al., 2022; Levante et al., 2024). Conversely, physical activity has been identified as a crucial factor in safeguarding both physical and psychological wellbeing (Bull et al., 2020; Qiu et al., 2025). However, the majority of students do not meet the recommended levels of physical activity (Ruiz et al., 2011; Brunet et al., 2013). This phenomenon has the potential to contribute to the development of adverse lifestyles and mental health challenges. In this context, psychological resilience is defined as a dynamic concept that increases an individual's capacity to cope with stress and supports characteristics such as self-control. This concept can be strengthened by physical activity (Qiu et al., 2025; Rutter, 2023). The extant research indicates that regular physical activity can support students' mental health by increasing psychological resilience and self-control levels (Fleshner, 2005; Ouergui et al., 2020; Micheletti Cremasco et al., 2021; Stanković et al., 2022). The present study hypothesizes that the minor impact observed on the subdimensions of emotional expression can be attributed to their common presence within the same social environment.

Research conducted in relation to judo has predominantly centered on professional or experienced athletes. These individuals have been documented to exhibit elevated levels of psychological resilience (Garrido-Muñoz et al., 2024). Conversely, the present study's focus on sedentary university students offers a distinctive contribution to the existing literature by demonstrating that judo training can have beneficial effects on mental health even at the introductory level. Moreover, the utilization of random sampling methods in the examination of judo, a widely practiced combat sport, contributes to the study's methodological rigor. The findings suggest that educational institutions and families should encourage young male university students to engage in structured physical activities such as judo. While these activities do not have a substantial impact on individuals' emotion expression skills, they have the potential to contribute to the development of psychological resilience and self-control skills.

This study has several limitations. The limited sample size precluded the ability to generalize the findings. The research focused on a 6-week time period and did not assess long-term outcomes, which limits the understanding of sustained impacts. The participants were exclusively drawn from a single department of a single university, a limitation that renders the results' generalizability to other student populations uncertain. The study did not address potential gender differences that could affect the results. Notwithstanding this limitation, the reliability of the results is ensured by the utilization of fully validated instruments adapted to the sample. In the future, researchers should endeavor to include larger and more diverse samples in their studies. Furthermore, they should examine long-term effects, address gender as a variable, and explore a wider range of Judo exercise protocols. These efforts will provide a more comprehensive understanding of the subject.

## Conclusions

This study offers substantial evidence that integrating Judo martial arts exercises into the academic curriculum of university students results in significant enhancements in various domains of sport psychology and mental health. The present findings suggest that judo martial arts exercises in a non-agonistic environment at the beginner level improve levels of psychological resilience, self-control, and emotion expression. These results underscore the critical role of judo martial arts training in the optimization of mental health and sport psychology. The observed enhancements in psychological resilience and self-control levels, in conjunction with regulated levels of emotional expression, suggest that judo exercises make a valuable contribution to mental health and sport psychology.

## Data Availability

The datasets presented in this study can be found in online repositories. The names of the repository/repositories and accession number(s) can be found in the article/supplementary material.
